# Prevalence and Treatment of Diarrhea Among Children in India, 2016-2021

**DOI:** 10.1001/jamanetworkopen.2025.26979

**Published:** 2025-08-14

**Authors:** Anoop Jain, Rockli Kim, S. V. Subramanian

**Affiliations:** 1 Department of Environmental Health, Boston University School of Public Health, Boston, Massachusetts; 2 Division of Health Policy and Management, College of Health Science, Korea University, Seoul, South Korea; 3 Interdisciplinary Program in Precision Public Health, Department of Public Health Sciences, Graduate School of Korea University, Seoul, South Korea; 4 Harvard Center for Population and Development Studies, Cambridge, Massachusetts; 5 Department of Social and Behavioral Sciences, Harvard T.H. Chan School of Public Health, Boston, Massachusetts

## Abstract

**Importance:**

The World Health Organization recommends the use of oral rehydration solution (ORS) for the treatment of child diarrhea. In India, adherence to these guidelines remains far less than complete.

**Objective:**

To elucidate geographic variations in ORS treatment for child diarrhea between and within India’s 720 districts.

**Design, Setting, and Participants:**

This repeated cross-sectional study used data from 2 rounds of India’s National Family Health Survey from 2016 and 2021. Complete diarrhea data were available from 246 695 children in 2016 and 223 785 children in 2021 from all 720 districts in both waves. Data on ORS treatment were available for 22 413 children in 2016 and 15 302 children in 2021. These children lived in 716 of India’s 720 districts in 2016 and in 718 districts in 2021. The data were analyzed in October 2024.

**Main Outcomes and Measures:**

The primary outcome was the district-level percentage of children who received ORS treatment if they had diarrhea in the past 2 weeks. The prevalence of diarrhea was also estimated. These values were estimated for both outcomes in 2016 and 2021 so that change over time could be estimated. The SD of the community-level prevalence of children who received ORS treatment by each district as a measure of the within-district inequality was also estimated for 2016 and 2021.

**Results:**

The study included 246 695 children younger than 5 years (mean [SD] age, 2.0 [1.4] years; 128 098 boys and 118 597 girls) for diarrhea prevalence and 22 413 children younger than 5 years (mean [SD] age, 1.5 [1.3] years; 12 050 boys and 10 363 girls) for ORS treatment in 2016. The study also included 223 785 children younger than 5 years (mean [SD] age, 2.0 [1.4] years; 115 632 boys and 108 153 girls) for diarrhea prevalence and 15 302 children younger than 5 years (mean [SD] age, 1.6 [1.4] years; 8160 boys and 7142 girls) for ORS treatment in 2021. Of the 9.2% (95% CI, 9.1%-9.3%) of children who had diarrhea in the 2 weeks prior to the survey in 2016, 50.6% (95% CI, 50.0%-51.3%) were treated with ORS. In 2021, 7.3% (95% CI, 7.2%-7.4%) of children had diarrhea, of whom 60.7% (95% CI, 59.9%-61.5%) were treated with ORS. There was considerable between-district variation in the prevalence of ORS treatment for child diarrhea (as of 2021, the state or Union Territory with the lowest district-level prevalence of ORS treatment was Haryana [49.0%]; the state or Union Territory with the highest district-level prevalence of ORS treatment was Jammu and Kashmir [83.4%]). The prevalence of ORS treatment decreased by more than 2.49 percentage points in 185 districts between 2016 and 2021 even though, nationally, the prevalence of ORS treatment increased over the same period, from a median of 57.5% (IQR, 47.2%-66.5%) to a median of 62.4% (IQR, 56.9%-72.5%). Finally, many districts with high prevalence of child diarrhea had low prevalence of ORS treatment. And in 71 districts, the prevalence of child diarrhea increased while the prevalence of ORS treatment decreased between 2016 and 2021.

**Conclusions and Relevance:**

This cross-sectional study of the prevalence of child diarrhea and its treatment with ORS in India found that the prevalence of ORS treatment for child diarrhea increased at a national level between 2016 and 2021, but this increase was not even between or within India’s 720 districts. The findings suggest that efforts aimed at promoting ORS treatment for child diarrhea in India must address the place-based factors that might be associated with underuse of this treatment.

## Key Points

**Question:**

To what extent does the use of oral rehydration solution (ORS) as treatment for child diarrhea vary between and within India’s 720 districts?

**Findings:**

In this repeated cross-sectional study, the prevalence of ORS treatment for child diarrhea increased at a national level between 2016 and 2021. However, this increase was not even between or within India’s 720 districts.

**Meaning:**

This study suggests that efforts aimed at promoting ORS treatment for child diarrhea in India must address the place-based factors that might be associated with underuse of this treatment.

## Teaser

This cross-sectional study examines the variations in oral rehydration solution treatment for child diarrhea between and within India’s 720 districts between 2016 and 2021.

## Introduction

Globally, the number of deaths of children younger than 5 years caused by diarrhea has decreased considerably over the past several decades. In 1980, approximately 2.7 million children younger than 5 years died of diarrhea.^1^ By 2021, this number had decreased to 443 833, an 83% reduction.^1^ Much of this decrease is attributable to treatment of diarrhea with oral rehydration solution (ORS),^1^ a glucose-electrolyte solution that the World Health Organization (WHO) and UNICEF have long recommended as a primary treatment via oral rehydration therapy for all but the most severe cases of diarrhea.^2^

Despite being easy to use and highly effective, the rate of ORS treatment varies considerably across regions. The prevalence of ORS use was 71.8% in Namibia in 2013, 66.4% in Zambia in 2018, and 63.8% in Malawi in 2016.^3^ However, the prevalence of ORS use was 19.2% in Cameroon in 2011 and 19.7% in Chad in 2015. The prevalence of ORS treatment also varies considerably subnationally, which is especially true in low- and middle-income countries (LMICs).^4^ Treatment with ORS was below 50% in most subnational administrative units in 94 LMICs as of 2017.^4^

Variations in ORS treatment for child diarrhea has been found across India’s states and Union Territories (UTs).^5^ As of 2021, approximately 61% of all children in India with diarrhea received ORS treatment.^6^ However, as of 2021, the extent to which children were receiving ORS treatment for diarrhea varied between states and UTs. Oral rehydration solution treatment for diarrhea was 45% in Goa and 88% in Dadra and Nagar Haveli and in Daman and Diu.

What remains unknown, however, is the extent to which the provision of ORS treatment varies across and within India’s districts. Each Indian state or UT is subdivided into districts, led by a District Magistrate or District Collector. These individuals oversee the prioritization and implementation of programs launched by India’s central government aimed at ensuring that the needs of a district’s citizens are met.^7,8^ Thus, many health outcomes depend on the performance of district administrators. In the context of child diarrhea, prior research shows that the prevalence of child diarrhea varied between India’s districts. In 2016, districts in Uttar Pradesh, Haryana, and Maharashtra had the highest rates of child diarrhea.^9^ Another study showed that as of 2021, the prevalence of diarrhea was higher than the national mean in 33% of India’s districts and that districts with high rates of child diarrhea were located in Maharashtra, Bihar, Odisha, and Gujarat.^10,11^

A comprehensive study of district-level variations in ORS treatment across all of India’s districts has not been done, to our knowledge. Previous studies have shown that some districts in states such as Gujarat, Uttar Pradesh, and Bihar have required interventions that aim to increase ORS treatment coverage.^12,13^ How ORS treatment coverage has changed over time and how this corresponds with changes in the district-level prevalence of diarrhea are far less understood. Filling this gap can help policymakers understand which districts require additional support and can help them design contextually relevant interventions aimed at increasing ORS treatment coverage.

The purpose of this article is to elucidate changes in ORS treatment coverage across all of India’s 720 districts. In India, policies made at the national and state levels are implemented by district administrations, making these important geographic units to study.^14^ We also examined changes in within-district inequality in ORS treatment and how changes in the district-level prevalence of diarrhea correspond with changes in district-level prevalences of ORS treatment. We used data from 2 consecutive rounds of India’s National Family Health Survey (NFHS) from 2016 and 2021 for this study. Conducting this analysis is important given that diarrhea remains a considerable cause of mortality among children younger than 5 years in India.^15^

## Methods

### Data and Sampling Strategy

We used child diarrhea and ORS treatment data from NFHS-4 (2015-2016) and NFHS-5 (2019-2021). These Demographic and Health Surveys (DHSs) were designed to collect data on population health, nutrition, well-being, and socioeconomic status.^16^ In both rounds of the NFHS, clusters, which were villages in rural communities and wards in urban communities, were selected first with probability proportional to size within districts. Households were then randomly selected from villages and wards. The latest NFHS report provides a complete description of the sampling strategy.^3^ The institutional review board for the International Institute for Population Sciences approved data collection for the NFHS in 2016 and 2021 and determined that this analysis did not meet the criteria for human participants. This study followed the Strengthening the Reporting of Observational Studies in Epidemiology (STROBE) reporting guideline for cross-sectional studies.

### Study Population

Both NFHS-4 and NFHS-5 surveyed mothers about their live births in the previous 5 years. We included all living children aged 59 months or younger with complete data on 2-week diarrhea and ORS treatment from both surveys. NFHS-4 contains data from 259 627 children, of whom 247 743 were alive at the time of survey. Of these living children, 247 181 (99.8%) had complete information on diarrhea in the past 2 weeks. Data were missing (reported as “do not know”) for only 562 children. However, we lost information from an additional 486 children due to changes in district geometry, and we thus had complete information on diarrhea in the previous 2 weeks for 246 695 children. There were 22 500 children who had diarrhea in the previous 2 weeks, and we had complete ORS treatment data from 22 450 (99.8%). Only 50 children had missing data (reported as “do not know”) on ORS treatment. However, we lost information from an additional 37 children due to the changes in district geometry, and thus we had complete ORS information for 22 413 children.

NFHS-5 contains data from 232 920 children, of whom 224 218 were alive. Of these living children, 223 785 (99.8%) had complete information on diarrhea in the past 2 weeks. Data were missing (reported as “do not know”) for only 433 children. There were 15 334 children who had diarrhea in the previous 2 weeks, and we had complete ORS treatment data from 15 302 (99.8%). Only 32 children had missing data (reported as “do not know”) for ORS treatment.

### Outcomes

The WHO states that the primary treatment for diarrhea should be ORS and that zinc could help shorten the duration of diarrhea episodes.^17^ As such, the outcome of interest in this study was ORS treatment, as this is the primary treatment recommended by the WHO. Mothers with eligible children who reported their child as having diarrhea in the previous 2 weeks were asked whether the child was given ORS treatment for that episode of diarrhea. Mothers could respond “yes,” “no,” or “I do not know.” We also examined the prevalence of 2-week caregiver-reported diarrhea among children aged 59 months or younger. Mothers were asked whether their child had at least 1 episode of diarrhea in the past 2 weeks, to which they could respond “yes,” “no,” or “I do not know.”

### District Geometry

The goal of this study is to elucidate changes in the prevalence of ORS treatment for child diarrhea both between and within districts from 2016 to 2021. Although there were 640 districts at the time of NFHS-4, there were 707 districts when NFHS-5 was administered. These subnational boundary changes are common in India. However, to compare district-level changes in ORS treatment between 2016 and 2021, we used an updated geometry of 720 districts. We relied on this geometry instead of the 707-district geometry because in 2022, the state of Andhra Pradesh created 13 new districts. None of Andhra Pradesh’s districts from NFHS-5 match with the state’s updated district boundaries. This difference would not allow us to meaningfully interpret any results from the districts within Andhra Pradesh.

Andhra Pradesh’s new district geometry was included by first linking Assembly Constituency boundaries with the 707 districts from NFHS-5 as provided by the DHS spatial data repository,^18^ while the Assembly Constituency linkage information was provided by Andhra Pradesh’s Chief Electoral Officer.^19^ We linked to the Assembly Constituency boundaries because they are contained within each district boundary, allowing them to be used to create new district boundaries for Andhra Pradesh after dissolving the Assembly Constituency polygons.^19^ We used the Survey of India to ensure that the new 720-district shapefile had the same external boundary of India as per their specifications.^20^

As a part of this updated 720-district geometry, there were 577 unchanged districts from NFHS-4 and 694 unchanged districts from NFHS-5. Thus, clusters from these districts could be used directly to make the linkage. Using the cluster global positioning system coordinates, we spatially joined the clusters from the unmatched districts from NFHS-4 and NFHS-5 to assign them to a district in the updated 720-district shapefile. However, 54 clusters from 2016 were not assigned to new districts as we were unable to ascertain their location in one of the new districts.

### Statistical Analysis

Statistical analysis was performed in October 2024. We estimated a multilevel model in which children were nested in clusters *j*, districts *k*, and states *l* to ascertain the district-level prevalence of ORS treatment and diarrhea. We did this using a Markov chain Monte Carlo (MCMC) procedure and using the runmlwin command in Stata, version 18 (StataCorp LLC).^21^ Only estimating quasi-likelihood approaches, such as second-order predictive quasi-likelihood (2PQL), can result in downwardly biased variance estimates with binary outcomes.^22,23^ In addition, methods using adaptive quadrature often fail when there are a large number of clusters per level, as is the case in this present analysis.^24^ On the other hand, MCMC approaches provide more reliable estimates when there are a high number of clusters per level, and do not tend to have downwardly biased variance estimates.^25^

Thus, we first fit our models using 2PQL for the ORS estimates and first-order marginal quasi-likelihood for the diarrhea prevalence to obtain suitable starting values for the MCMC process.^26^ This was applied to the model logit (Pr*_ijkl_*) = β_0_ + (*u*_0_*_jkl_* + *v*_0_*_kl_* + *f*_0_*_l_*) for the ORS and diarrhea outcomes. In this model, β_0_ represents the constant, and *u*_0_*_jkl_*, *v*_0_*_kl_*, and *f*_0_*_l_* are the residual differentials for clusters *j*, districts *k*, and states *l*, respectively. We then specified the MCMC procedure with a burn-in of 1000 cycles and monitoring of 75 000 chains. For diarrhea, we specified a burn-in of 500 cycles and monitoring of 75 000 chains. These combinations allowed us to obtain effective sample sizes greater than 250, the number of independent samples equivalent to the number of dependent MCMC samples (75 000 in this case).^27,28^ The residuals for both ORS treatment and diarrhea were then used in the equation exp [β_0_ + (*u*_0_*_jkl_* + *v*_0_*_kl_* + *f*_0_*_l_*)]/[1 + exp [β_0_ + (*u*_0_*_jkl_* + *v*_0_*_kl_* + *f*_0_*_l_*)] to calculate the precision-weighted estimates for the share of each outcome in each cluster. These cluster estimates were then used to produce district-level mean values of each outcome in each survey round. We also estimated the SD of the cluster-level estimates within each district as a measure of inequality in both survey rounds. The output from our MCMC models is shown in eTable 1 in Supplement 1.

## Results

### Sample Characteristics

In 2016, we analyzed 246 695 children younger than 5 years (mean [SD] age, 2.0 [1.4] years; 128 098 boys and 118 597 girls) for diarrhea prevalence (Table). Of those children, 22 413 (mean [SD] age, 1.5 [1.3] years; 12 050 boys and 10 363 girls) reported having diarrhea in the past 2 weeks and were thus analyzed for ORS treatment. In 2021, we analyzed 223 785 children younger than 5 years (mean [SD] age, 2.0 [1.4] years; 115 632 boys and 108 153 girls) for diarrhea prevalence. Of those children, 15 302 (mean [SD] age, 1.6 [1.4] years; 8160 boys and 7142 girls) reported having diarrhea in the past 2 weeks and were thus analyzed for ORS treatment. Of the 9.2% (95% CI, 9.1%-9.3%) of children who had diarrhea in the 2 weeks prior to the survey in 2016, 50.6% (95% CI, 50.0%-51.3%) were treated with ORS. In 2021, 7.3% (95% CI, 7.2%-7.4%) of children had diarrhea in the 2 weeks prior to the survey, of whom 60.7% (95% CI, 59.9%-61.5%) were treated with ORS. These weighted means are descriptive values from the sample, while all values presented hereafter were results from our model estimates.

**Table.** Weighted Mean Prevalence of Diarrhea and ORS Treatment Among Children in India Between 2016 and 2021^a^

State or Union Territory	Diarrhea prevalence				ORS treatment			
	2016		2021		2016		2021	
	No.	Mean (95% CI or district range)	No.	Mean (95% CI or district range)	No.	Mean (95% CI or district range)	No.	Mean (95% CI or district range)
All of India, mean (95% CI), No.	246 695	9.2 (9.1-9.3)	223 785	7.3 (7.2-7.4)	22 413	50.6 (50.0-51.3)	15 302	60.7 (59.9-61.5)
State, mean (district range), No.								
Andhra Pradesh	2980	5.4 (2.4-9.9)	2740	5.9 (2.6-12.1)	191	49.5 (38.6-64.3)	198	66.4 (47.2-79.1)
Arunachal Pradesh	4598	5.9 (3.0-10.0)	5414	4.7 (1.3-12.2)	310	68.8 (43.8-83.4)	308	66.5 (49.6-78.0)
Assam	9697	2.2 (1.1-4.9)	10 276	4.4 (1.6-8.0)	257	53.5 (36.2-70.6)	544	71.0 (54.1-81.6)
Bihar	24 064	8.6 (3.2-16)	19 912	11.4 (2.4-38.2)	2291	46.2 (27.4-61.8)	2486	58.6 (42.6-70.2)
Chhattisgarh	8696	7.3 (3.5-16.6)	8098	3.1 (0.9-14.8)	726	73.0 (55.8-84.9)	312	74.3 (58.7-83.6)
Goa	410	3.1 (1.8-4.4)	336	2.4 (2.3-2.5)	14	59.1 (58.7-59.2)	10	69.5 (68.5-71.0)
Gujarat	7399	7.7 (3.6-12.2)	9532	7.7 (2.4-14)	657	43.5 (29.1-64.3)	854	68.9 (50.8-84.0)
Haryana	7568	6.9 (2.4-12.0)	6634	4.1 (2.6-6.4)	588	61.5 (36.9-80.3)	336	49.0 (35.3-61.8)
Himachal Pradesh	2808	5.6 (3.4-9.0)	2560	4.4 (2.1-9.0)	184	62.8 (51.7-75.0)	139	71.9 (63.6-80.4)
Jharkhand	11 625	5.9 (2.7-15.3)	9634	6.2 (2.6-12.6)	792	45.4 (31.2-58.3)	701	59.7 (33.6-76.8)
Karnataka	7533	3.8 (1.5-7.6)	8140	4.6 (2.2-7.6)	340	60.1 (44.8-77.0)	460	72.5 (63.1-86.5)
Kerala	2443	2.9 (1.7-4.1)	2707	3.0 (1.4-6.8)	85	53.7 (40.0-65.5)	102	63.5 (54.0-74.4)
Madhya Pradesh	23 210	8.5 (3.9-16.6)	15 516	5.1 (2.1-9.5)	2211	57.2 (35.5-75.1)	954	68.6 (44.2-85.2)
Maharashtra	9146	7.9 (3.8-13.9)	9252	8.6 (2.7-17.0)	828	61.6 (48.8-77.4)	920	59.7 (44.6-78.5)
Manipur	5477	4.8 (2.5-6.9)	3128	4.1 (2.1-8.7)	308	60.4 (47.5-65.3)	153	66.2 (57.6-78.3)
Meghalaya	4261	10.5 (2.7-26.1)	6368	8.9 (5.0-12.6)	487	82.3 (66.1-94.2)	628	75.7 (68.7-86.4)
Mizoram	4662	4.6 (2.8-7.8)	2400	2.8 (1.2-5.6)	253	67.1 (53.1-76.3)	82	72.5 (62.3-78.6)
Nagaland	4410	4.6 (1.7-8.1)	2933	3.2 (1.4-5.3)	236	44.2 (30.7-53.5)	115	57.8 (40.0-75.2)
Odisha	10 575	8.2 (3.0-18.8)	8153	7.3 (2.7-15.0)	985	72.9 (61.3-85.8)	699	69.8 (55.9-81.5)
Punjab	5010	6.1 (3.4-14.4)	5400	3.9 (1.6-9.6)	361	70.6 (54.1-81.6)	259	60.3 (43.1-73.0)
Rajasthan	16 065	6.3 (2.5-11.1)	14 138	5.3 (2.3-13.4)	1164	58.3 (37.3-77.0)	891	64.3 (45.7-87.5)
Sikkim	975	1.9 (1.2-2.2)	605	4.1 (2.9-4.7)	19	63.3 (55.7-71.7)	31	62.9 (54.8-70.9)
Tamil Nadu	7716	6.9 (4.1-12.8)	6370	2.9 (1.5-6.3)	624	61.8 (40.8-73.5)	237	55.5 (35.5-70.3)
Telangana	2348	7.4 (3.9-12.8)	7100	6.9 (2.3-13.0)	201	57.7 (36.8-71.8)	583	56.6 (37.7-73.8)
Tripura	1291	4.3 (3.2-5.6)	1986	5.3 (2.0-8.6)	65	51.1 (37.1-62.8)	128	65.6 (47.1-77.3)
Uttar Pradesh	38 720	14.0 (4.6-27.7)	33 786	4.7 (1.7-12.7)	5845	36.1 (19.4-58.3)	1904	52.2 (28.0-72.8)
Uttarakhand	5561	16.1 (13.9-19.7)	3644	3.6 (2.2-4.7)	972	56.1 (36.7-68.8)	163	54.6 (41.7-64.0)
West Bengal	5152	5.2 (2.6-8.1)	5479	5.6 (2.4-9.8)	315	66.4 (50.7-79.6)	368	77.7 (66.0-87.1)
Union Territory								
Andaman and Nicobar Islands	637	4.2 (3.9-4.7)	441	4.6 (4.1-4.8)	32	79.5 (75.1-83.7)	24	79.0 (72.9-85.3)
Chandigarh	187	4.2 (4.2-4.2)	169	3.2 (3.2-3.2)	9	59.4 (59.4-59.4)	6	62.4 (62.4-62.4)
Dadra and Nagar Haveli and Daman and Diu	706	4.3 (2.5-6.6)	772	2.3 (1.4-3.5)	35	74.9 (58.5-81.1)	20	82.0 (81.0-82.7)
Jammu and Kashmir	7253	9.5 (2.5-40.4)	5750	4.5 (1.4-8.5)	753	72.8 (52.0-81.5)	318	83.4 (71.4-90.5)
Ladakh	663	3.3 (2.8-3.7)	515	7.1 (5.0-9.2)	21	74.4 (70.1-76.1)	45	77.2 (74.8-81.4)
Lakshadweep	300	4.6 (4.6-4.6)	276	2.1 (2.1-2.1)	16	54.0 (54.0-54.0)	6	59.9 (59.9-59.9)
NCT of Delhi	1488	9.3 (5.5-15.8)	2852	9.0 (6.1-11.9)	160	68.6 (57.9-74.2)	301	67.2 (56.5-82.9)
Puducherry	1061	6.3 (3.4-11.9)	757	2.0 (1.2-3.4)	78	64.2 (56.7-67.0)	17	56.2 (38.1-63.5)

Abbreviations: NCT, National Capital Territory; ORS, oral rehydration solution.

^a^ The state values (district mean and district minimum and maximum) were derived from our multilevel models. The all-India values are the weighted means from the sample.

**Figure 1.**
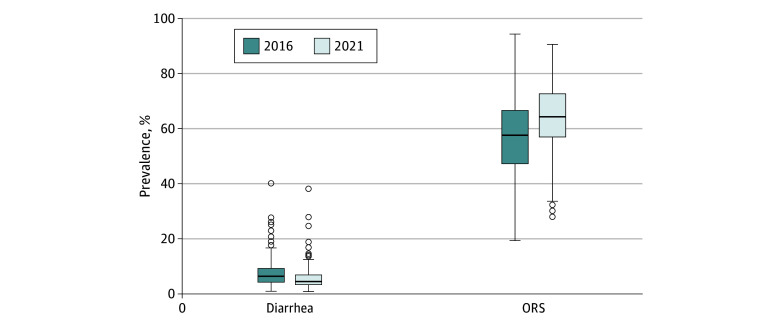
Distribution of District-Level Prevalence of Diarrhea and Oral Rehydration Solution (ORS) Treatment in 2016 and 2021 The upper and lower whiskers represent minimum and maximum values, respectively. The upper outline of the box depicts the 75th percentile, and the lower outline the 25th percentile. The solid line within the box shows the median (50th percentile). The dots represent outliers.

**Figure 2.**
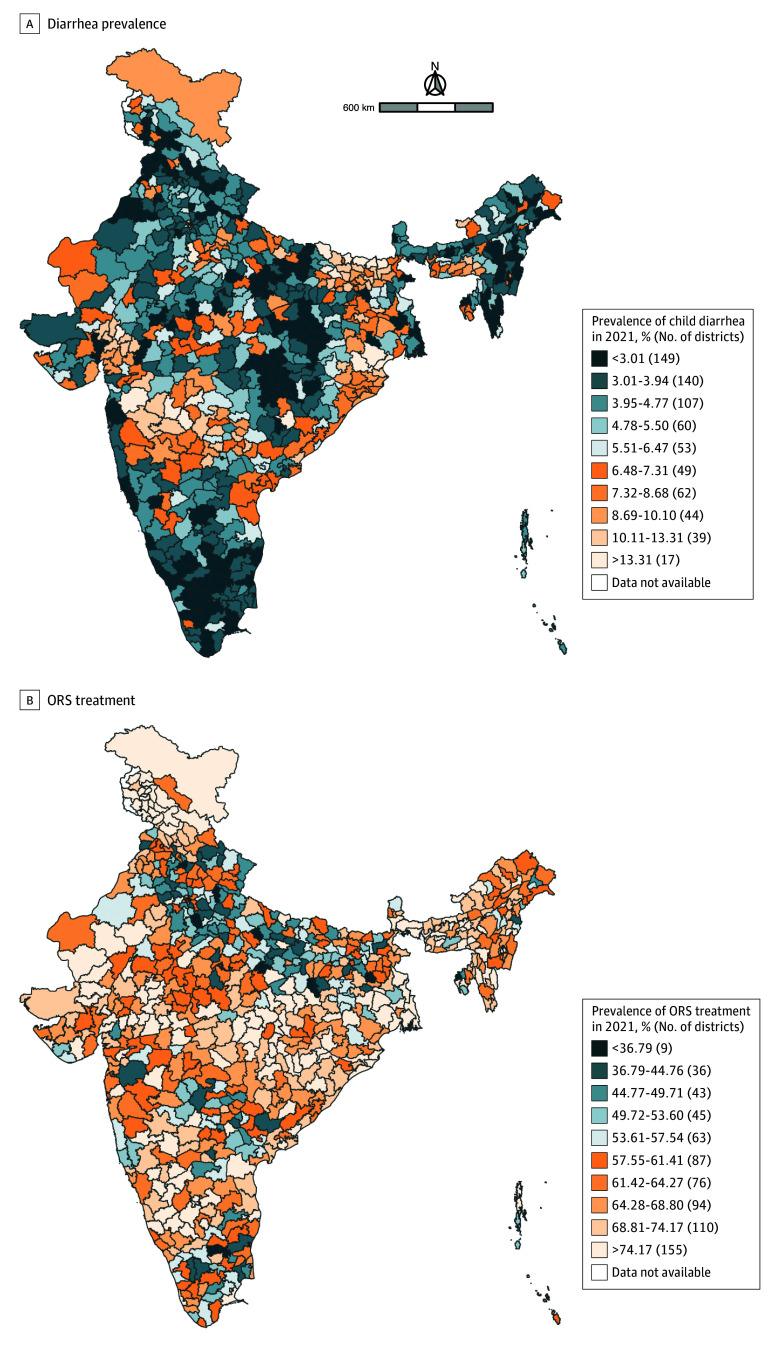
Maps Depicting the District-Level Prevalence of Child Diarrhea and Oral Rehydration Solution (ORS) Treatment Decile cutoff values are based on the prevalence of each outcome in 2016 to highlight changes in district prevalence between the 2 time periods.

Children with complete ORS data lived in 716 of India’s 720 districts in 2016 and 718 of India’s 720 districts in 2021. The median district prevalence of ORS treatment in 2016 was 57.5% (IQR, 47.2%-66.5%), which increased to 64.2% (IQR, 56.9%-72.5%) in 2021 (Figure 1). The increase in ORS treatment corresponded with a decrease in the district-level prevalence of diarrhea between 2016 and 2021. As of 2021, the state or UT with the lowest district-level prevalence of ORS treatment was Haryana (49.0%), while the state or UT with the highest district-level prevalence of ORS treatment was Jammu and Kashmir (83.4%) (Table and Figure 2). The mean district-level prevalence of ORS treatment was 68.9% in Gujarat compared with 58.6% in Bihar and 52.2% in Uttar Pradesh (Table and Figure 2). In 2021, the state or UT with the lowest district-level mean of child diarrhea was Puducherry (2.0%), and the state or UT with the highest district-level mean of child diarrhea was Bihar (11.4%) (Table and Figure 2).

### Changes in District Prevalence of ORS Treatment

**Figure 3.**
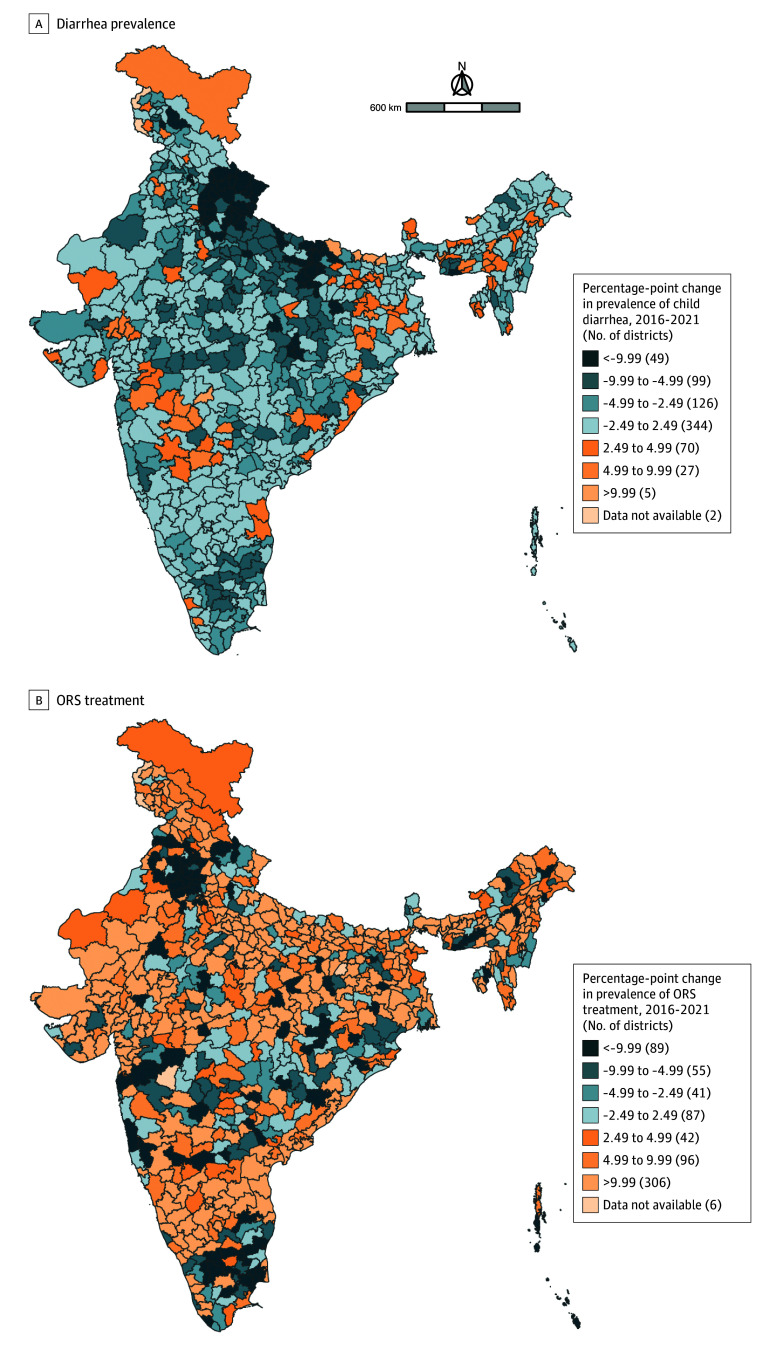
Maps Depicting Changes in District-Level Prevalence of Diarrhea and Oral Rehydration Solution (ORS) Treatment Between 2016 and 2021

We found that the prevalence of ORS treatment for children with diarrhea decreased by more than 9.99 percentage points in 89 districts between 2016 and 2021 (Figure 3). The prevalence of ORS treatment for children with diarrhea decreased between 4.99 and 9.99 percentage points in 55 districts between 2016 and 2021. The prevalence of ORS treatment among children with diarrhea decreased between 2.49 and 4.99 percentage points in 41 districts, and the prevalence of ORS treatment for children with diarrhea changed between −2.49 and 2.49 percentage points in 87 districts from 2016 to 2021. The prevalence of ORS treatment among children with diarrhea increased between 2.49 and 4.99 percentage points in 42 districts, and it increased between 4.99 and 9.99 percentage points in 96 districts from 2016 to 2021. Finally, the prevalence of ORS treatment for child diarrhea increased more than 9.99 percentage points in 306 districts between 2016 and 2021.

We found that, between 2016 and 2021, there were 318 districts in which the district-level prevalence of ORS treatment increased, but within-district, between-community inequality worsened (eTable 2 and eFigure 1 in Supplement 1). We also found that in 190 districts, both the district-level prevalence of ORS treatment and the within-district, between-community inequality worsened. In 32 districts, the district-level prevalence of ORS treatment decreased, while there was a decrease in within-district, between-community inequality. Finally, the district-level prevalence of ORS treatment and the within-district, between-community inequality improved in 162 districts between 2016 and 2021.

### District Correlations Between Child Diarrhea and ORS Treatment

**Figure 4.**
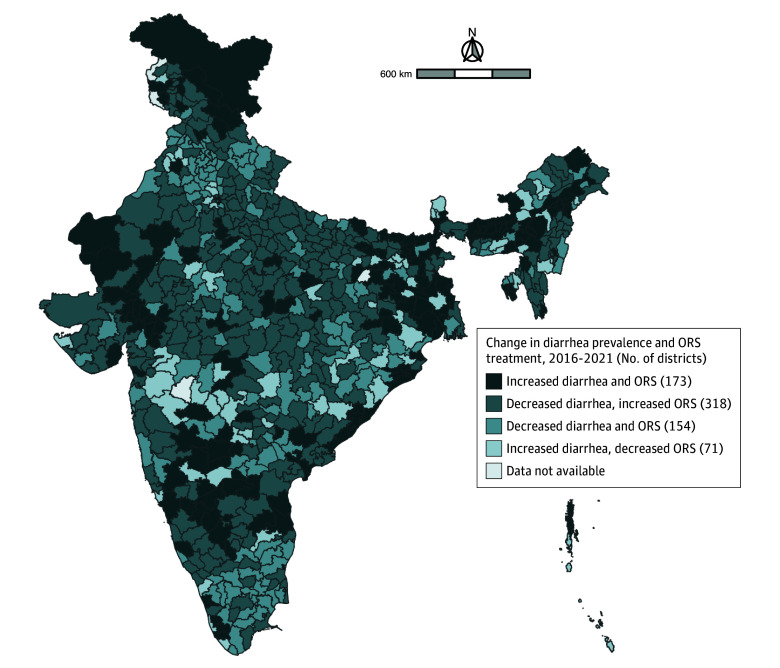
Map Depicting Changes in the Prevalence of Diarrhea and Oral Rehydration Solution (ORS) Treatment by District Between 2016 and 2021

Between 2016 and 2021, 173 districts experienced an increase in both diarrhea prevalence and the prevalence of ORS treatment (Figure 4). We found that in 318 districts, the prevalence of diarrhea decreased while the prevalence of ORS treatment increased between 2016 and 2021. In 154 districts, the prevalence of diarrhea decreased as did the prevalence of ORS treatment between 2016 and 2021. Finally, between 2016 and 2021, 71 districts experienced an increase in diarrhea prevalence while the prevalence of ORS treatment decreased.

We found that in 2021, the mean district-level prevalence of child diarrhea was 5.5% and that the mean district-level prevalence of ORS treatment was 64.1% (eFigure 2 in Supplement 1). As of 2021, the prevalence of diarrhea and the prevalence of ORS treatment were above these mean values in 136 districts. In 225 districts, the prevalence of diarrhea was below the district-level mean value, while the prevalence of ORS treatment was above the district-level mean value. We found that there were 226 districts in which the prevalence of diarrhea and the prevalence of ORS treatment were below the district-level mean values. Finally, there were 131 districts in which the prevalence of diarrhea was above the district-level mean value, while the prevalence of ORS treatment was below the district-level mean value. However, our correlation analysis showed very weak associations between the district-level prevalence of diarrhea and ORS treatment in both 2016 and 2021 (eFigure 3 in Supplement 1).

## Discussion

This study had 4 salient findings. First, as of 2021, we found considerable between-district variation in the prevalence of ORS treatment for child diarrhea (the state or UT with the lowest district-level prevalence of ORS treatment was Haryana [49.0%]; the state or UT with the highest district-level prevalence of ORS treatment was Jammu and Kashmir [83.4%]). Second, we found that the prevalence of ORS treatment decreased more than 2.49 percentage points in 185 districts between 2016 and 2021 even though, nationally, the prevalence of ORS treatment increased from 2016 to 2021. In 89 of these districts, the prevalence of ORS treatment decreased by more than 9.99 percentage points from 2016 to 2021. Third, we found that the district-level mean and within-district inequality in ORS treatment worsened in 190 districts between 2016 and 2021. In other words, the ORS treatment gap widened between communities while the prevalence of ORS treatment decreased within 190 of India’s districts. Fourth, despite us not finding strong correlations between child diarrhea prevalence and ORS treatment prevalence, many of India’s districts with a high prevalence of child diarrhea had low prevalence of ORS treatment and between 2016 and 2021, 71 of India’s districts experienced increases in child diarrhea but decreases in the prevalence of ORS treatment.

Our findings are policy relevant for several reasons. Although ORS is widely considered the primary treatment option for child diarrhea, except for the most severe cases, adherence to this recommendation remains far less than perfect, creating a large know-do gap.^29^ Previous explanations of this know-do gap in India focus on individual-level factors, such as prescriber behaviors or caregiver perceptions and knowledge of treatment options. One study from Ujjain in Madhya Pradesh showed that 71% of prescriptions for the treatment of child diarrhea included 1 or more antibiotics,^30^ a likely consequence of caregivers believing that antibiotics are most effective in stopping diarrhea.^31^ Another study conducted in 7 of India’s states found that caregivers feel that ORS dosing recommendations are impractical and thus tend to rely more on antibiotics.^31^ This has led to lower demand for ORS treatment^31,32^ despite its efficacy, and has led to interventions that seek to spur demand for ORS treatment through public health messaging campaigns.^32^ Evidence from Karnataka and Bihar also suggests that health care professionals perceive that patients do not want ORS, which leads to underprescribing.^33^ Maternal education is also an important factor associated with ORS treatment for child diarrhea,^34^ and rates of maternal education vary considerably throughout India.^3^ Women in Karnataka are more likely than women in Bihar to have completed 10th grade,^6^ which could also help explain why we found that districts in Karnataka had a higher prevalence of ORS treatment than districts in Bihar. Mothers also receive critical child health and nutrition information, including information pertaining to the treatment of diarrhea, from Anganwadi centers (part of the Indian public health care system that provide basic health care in a village). Providing proper information to mothers on the importance of ORS treatment for child diarrhea has been shown to sustain ORS use across episodes of child diarrhea.^35^

The results from our study, however, highlight a geographic distribution and burden of this know-do gap. As of 2021, there were 131 districts that had the highest prevalence of child diarrhea but the lowest prevalence of ORS treatment. We also found that between 2016 and 2021, 71 districts experienced an increase in diarrhea prevalence, while the prevalence of ORS treatment decreased. These findings highlight the contextual factors that might contribute to varying prevalence of ORS treatment.

There are several structural factors that might contribute to the geographic distribution of this know-do gap in ORS treatment of child diarrhea across India’s districts. For instance, previous work has shown that efforts aimed at scaling up ORS treatment were stifled by low availability of ORS or the materials needed to produce it.^36^ A higher share of child diarrhea cases were treated with ORS in Gujarat than in Uttar Pradesh or Bihar due to the uninterrupted supply of ORS in Gujarat.^37^ This could help explain our findings that showed that the mean district-level prevalence of ORS treatment was 68.9% in Gujarat, compared with 58.6% in Bihar and 52.2% in Uttar Pradesh. It is possible that these supply-side constraints vary within districts, too, a factor that could explain our finding that within-district, between-community inequality in ORS treatment worsened in many districts. Furthermore, previous research demonstrates that the quality of health care mothers receive varies between communities within districts throughout India and often depends on the availability of necessary prescriptions.^38^ Also, the lack of political commitment and instability have been identified as key reasons as to why ORS supply and distribution can often falter in India and other LMICs.^35,39^ Thus, our finding that ORS treatment varies across districts could be because the extent to which ORS treatment is prioritized varies by district administrations and officials, who are ultimately responsible for implementing policies.^14^

In India, as is the case in countries such as Nigeria, Bangladesh, and Thailand, approximately one-third of primary care visits are to health care professionals without formal medical training,^40^ an indication of the dearth of trained health care workers throughout the country.^41^ In LMICs, adequate clinician training has been recognized as a key factor in improving rates of ORS treatment for child diarrhea.^39,42^ However, in places such as rural Bihar, health care professionals in one study were unable to properly diagnose and treat diarrhea.^29^ It is possible that variations in health care professional training could explain the between-district differences in ORS treatment prevalences found in this study. District administrations should focus on strengthening the quality of their health care systems, which has been shown to improve ORS treatment prevalences for child diarrhea.^43^

### Limitations

This study has 3 data-related limitations. First, a 2-week recall period using DHS data can underestimate the burden of child diarrhea.^44^ Thus, it is possible that we underestimated the prevalence of diarrhea and ORS treatment in this study as well. Second, 54 clusters from 2016 were not included in the final analytic sample as they could not be matched with the new district geometry. Therefore, our results are not representative of those communities. Third, it is possible that the results from Andhra Pradesh are underpowered, as we included 26 districts from the state while data were collected from only 13 districts. However, previously published work shows small standard errors for outcome estimates from this state, suggesting that they are precise.^45^

## Conclusions

In this cross-sectional study of the prevalence of child diarrhea and its treatment with ORS in India, we found that the prevalence of ORS treatment for child diarrhea increased at a national level between 2016 and 2021. This increase was not even between or within India’s 720 districts. Although previous studies attributed the know-do gap in ORS treatment to individual-level attitudes, behaviors, and perceptions of prescribers and caregivers, our findings highlighted that place-based factors might also play an important role in shaping this gap between the incidence of child diarrhea and ORS treatment. Our results underscored that policies aimed at promoting ORS treatment for child diarrhea should consider the myriad contextual factors between and within districts.

## Notes

Supplement 1. **eTable 1.** Output Estimates of Models Used to Derive District-Level Prevalence of Child Diarrhea and ORS Treatment in 2016 and 2021 **eTable 2.** Number of Districts by State for Which the Outcome Prevalence and Inequality Worsened and/or Improved Over Time. **eFigure 1.** Changes in District Mean ORS Treatment and Within-District Between-Community Inequality of ORS Treatment **eFigure 2.** Map Depicting the District-Level Correlation Between Diarrhea Prevalence and ORS Treatment in 2021 **eFigure 3.** Scatter Plots Describing the Correlation Between the District-Level Prevalence of Diarrhea and ORS Treatment in 2016 and 2021

Supplement 2.
Data Sharing Statement
