# Supplementary material for: Prevalence and Treatment of Diarrhea Among Children in India, 2016-2021
Source: JAMA Netw Open. 2025 Aug 14;8(8):e2526979. doi: 10.1001/jamanetworkopen.2025.26979 (PMC12355290; doi:10.1001/jamanetworkopen.2025.26979)
Supplement: Supplement 1. — eTable 1. Output Estimates of Models Used to Derive District-Level Prevalence of Child Diarrhea and ORS Treatment in 2016 and 2021 eTable 2. Number of Districts by State for Which the Outcome Prevalence and Inequality Worsened and/or Improved Over Time. eFigure 1. Changes in District Mean ORS Treatment and Within-District Between-Community Inequality of ORS Treatment eFigure 2. Map Depicting the District-Level Correlation Between Diarrhea Prevalence and ORS Treatment in 2021 eFigure 3. Scatter Plots Describing the Correlation Between the District-Level Prevalence of Diarrhea and ORS Treatment in 2016 and 2021 [file jamanetwopen-e2526979-s001.pdf]

Supplementary Online Content

Jain A, Kim R, Subramanian SV. Prevalence and treatment of diarrhea among children in India, 2016-2021. *JAMA Netw Open.* 2025;8(8):e2526979. doi:10.1001/jamanetworkopen.2025.26979

eTable 1. Output Estimates of Models Used to Derive District-Level Prevalence of Child Diarrhea and ORS Treatment in 2016 and 2021
eTable 2. Number of Districts by State for Which the Outcome Prevalence and Inequality Worsened and/or Improved Over Time.
eFigure 1. Changes in District Mean ORS Treatment and Within-District Between-Community Inequality of ORS Treatment
eFigure 2. Map Depicting the District-Level Correlation Between Diarrhea Prevalence and ORS Treatment in 2021
eFigure 3. Scatter Plots Describing the Correlation Between the District-Level Prevalence of Diarrhea and ORS Treatment in 2016 and 2021

This supplementary material has been provided by the authors to give readers additional information about their work.

© 2025 Jain A et al. *JAMA Network Open.*

eTable 1. Output Estimates of Models Used to Derive District-Level Prevalence of Child Diarrhea and ORS Treatment in 2016 and 2021

	Descriptives				1MQL			MCMC					
**2016 Diarrhea**	**n**	minimum	average	maximum	coef	Std Err	95% CI	coef	Std Dev	ESS	95% CI	Other MCMC	
**Constant**					- 2.51	0.08	(-2.66 to - 2.35)	- 2.89	0.08	28794	(-3.06 to -2.72))	dbar	134397.2
**State**	36	187	6866.1	38852	0.18	0.05	(0.08 - 0.28)	0.23	0.07	15254	(0.13 - 0.39)	thetabar	128080.1
**District**	640	117	386.2	1268	0.28	0.02	(0.24 to 0.32)	0.20	0.02	11270	(0.17 - 0.23)	effective no. of pars	6317.1
**Cluster**	28321	1	8.7	42	0.88	0.02	(0.84 to 0.92)	0.50	0.02	864	(0.47 - 0.54)	B DIC	140714.3
**2016 ORS**					**2PQL**			**MCMC**					
**Constant**					0.44	0.09	(0.26 - 0.61)	0.46	0.10	28220	(0.27 - 0.65)	dbar	25723.3
**State**	36	9	623.6	5870	0.22	0.06	(0.09 - 0.34)	0.26	0.08	14281	(0.14 - 0.47)	thetabar	22811
**District**	639	1	35.1	306	0.17	0.02	(0.12 - 0.21)	0.19	0.02	2680	(0.14 - 0.24)	effective no. of pars	2912.2
**Cluster**	12144	1	1.8	16	0.47	0.04	(0.40 - 0.55)	0.82	0.07	362	(0.69 - 0.96)	B DIC	28635.5
**2021 Diarrhea**					**1MQL**			**MCMC**					
**Constant**					- 2.72	0.07	(-2.85 to -2.59)	- 3.14	0.07	16912	(-3.29 to -3.00)	dbar	98843.1
**State**	36	169	6216.2	33768	0.12	0.04	(0.05 - 0.20)	0.16	0.05	7819	(0.08 - 0.28)	thetabar	93200.5
**District**	707	57	316.5	1076	0.29	0.02	(0.25 - 0.33)	0.24	0.02	8405	(0.21 - 0.28)	effective no. of pars	5642.6
**Cluster**	29757	1	7.5	43	0.92	0.03	(0.87 - 0.97)	0.62	0.03	550	(0.58 - 0.68)	B DIC	104485.7
**2021 ORS**					**2PQL**			**MCMC**					
**Constant**					0.68	0.08	(0.53 - 0.83)	0.73	0.09	19509	(0.56 - 0.90)	dbar	16698.6
**State**	36	6	425.1	2486	0.15	0.05	(0.06 - 0.24)	0.19	0.07	11098	(0.10 - 0.36)	thetabar	14129.6
**District**	705	1	21.7	254	0.15	0.02	(0.10 - 0.19)	0.17	0.03	1188	(0.11 - 0.24)	effective no. of pars	2569.1
**Cluster**	9499	1	1.6	15	0.58	0.05	(0.49 - 0.68)	1.18	0.12	269	(0.95 - 1.44)	B DIC	19267.8

eTable 2. Number of Districts by State for Which the Outcome Prevalence and Inequality Worsened and/or Improved Over Time. Values in parentheses indicate the percentage of total districts by state in each category of improvement or worsening (indicated in the last row) for both outcomes

	Worsened mean & inequality		Improved mean, worsened inequality		Improved mean & inequality		Worsened mean, improved inequality	
**State**	**Diarrhea prevalence**	**ORS treatment**	**Diarrhea prevalence**	**ORS treatment**	**Diarrhea prevalence**	**ORS treatment**	**Diarrhea prevalence**	**ORS treatment**
Andhra Pradesh	13 (5.7%)	1 (0.5%)	5 (5.4%)	16 (5%)	7 (1.8%)	6 (3.7%)	1 (6.3%)	0 (0%)
Arunachal Pradesh	8 (3.5%)	8 (4.2%)	2 (2.2%)	8 (2.5%)	10 (2.6%)	1 (0.6%)	0 (0%)	1 (3.1%)
Assam	29 (12.6%)	2 (1.1%)	2 (2.2%)	21 (6.6%)	1 (0.3%)	9 (5.6%)	1 (6.3%)	0 (0%)
Bihar	22 (9.6%)	7 (3.7%)	1 (1.1%)	20 (6.3%)	11 (2.9%)	9 (5.6%)	4 (25%)	1 (3.1%)
Chhattisgarh	1 (0.4%)	12 (6.3%)	3 (3.3%)	9 (2.8%)	23 (6%)	6 (3.7%)	0 (0%)	0 (0%)
Goa	1 (0.4%)	1 (0.5%)	0 (0%)	0 (0%)	1 (0.3%)	1 (0.6%)	0 (0%)	0 (0%)
Gujarat	13 (5.7%)	4 (2.1%)	8 (8.7%)	15 (4.7%)	12 (3.1%)	14 (8.6%)	0 (0%)	0 (0%)
Haryana	4 (1.7%)	15 (7.9%)	4 (4.3%)	2 (0.6%)	14 (3.7%)	1 (0.6%)	0 (0%)	3 (9.4%)
Himachal Pradesh	4 (1.7%)	1 (0.5%)	0 (0%)	6 (1.9%)	8 (2.1%)	5 (3.1%)	0 (0%)	0 (0%)
Jharkhand	13 (5.7%)	5 (2.6%)	3 (3.3%)	17 (5.3%)	7 (1.8%)	1 (0.6%)	1 (6.3%)	1 (3.1%)
Karnataka	15 (6.5%)	2 (1.1%)	3 (3.3%)	14 (4.4%)	11 (2.9%)	14 (8.6%)	1 (6.3%)	0 (0%)
Kerala	7 (3%)	4 (2.1%)	1 (1.1%)	6 (1.9%)	6 (1.6%)	2 (1.2%)	0 (0%)	0 (0%)
Madhya Pradesh	6 (2.6%)	9 (4.7%)	5 (5.4%)	22 (6.9%)	40 (10.5%)	19 (11.7%)	0 (0%)	1 (3.1%)
Maharashtra	14 (6.1%)	18 (9.5%)	3 (3.3%)	10 (3.1%)	18 (4.7%)	5 (3.1%)	1 (6.3%)	2 (6.3%)
Manipur	3 (1.3%)	4 (2.1%)	0 (0%)	3 (0.9%)	6 (1.6%)	2 (1.2%)	0 (0%)	0 (0%)
Meghalaya	5 (2.2%)	7 (3.7%)	0 (0%)	3 (0.9%)	6 (1.6%)	1 (0.6%)	0 (0%)	0 (0%)
Mizoram	1 (0.4%)	1 (0.5%)	0 (0%)	4 (1.3%)	7 (1.8%)	3 (1.9%)	0 (0%)	0 (0%)
Nagaland	2 (0.9%)	0 (0%)	2 (2.2%)	9 (2.8%)	7 (1.8%)	1 (0.6%)	0 (0%)	0 (0%)
Odisha	10 (4.3%)	15 (7.9%)	4 (4.3%)	7 (2.2%)	13 (3.4%)	5 (3.1%)	3 (18.8%)	3 (9.4%)
Punjab	4 (1.7%)	12 (6.3%)	3 (3.3%)	6 (1.9%)	14 (3.7%)	2 (1.2%)	1 (6.3%)	2 (6.3%)
Rajasthan	11 (4.8%)	8 (4.2%)	10 (10.9%)	15 (4.7%)	12 (3.1%)	10 (6.2%)	0 (0%)	0 (0%)
Sikkim	4 (1.7%)	3 (1.6%)	0 (0%)	1 (0.3%)	0 (0%)	0 (0%)	0 (0%)	0 (0%)
Tamil Nadu	0 (0%)	13 (6.8%)	2 (2.2%)	7 (2.2%)	30 (7.9%)	4 (2.5%)	0 (0%)	7 (21.9%)
Telangana	7 (3%)	11 (5.8%)	14 (15.2%)	13 (4.1%)	9 (2.4%)	2 (1.2%)	1 (6.3%)	3 (9.4%)
Tripura	4 (1.7%)	2 (1.1%)	1 (1.1%)	3 (0.9%)	3 (0.8%)	2 (1.2%)	0 (0%)	1 (3.1%)
Uttar Pradesh	1 (0.4%)	7 (3.7%)	7 (7.6%)	53 (16.7%)	67 (17.5%)	10 (6.2%)	0 (0%)	4 (12.5%)
Uttarakhand	0 (0%)	6 (3.2%)	0 (0%)	4 (1.3%)	13 (3.4%)	2 (1.2%)	0 (0%)	1 (3.1%)
West Bengal	13 (5.7%)	1 (0.5%)	3 (3.3%)	11 (3.5%)	4 (1%)	7 (4.3%)	0 (0%)	1 (3.1%)
**Union Territory**								
Andaman & Nicobar Islands	2 (0.9%)	2 (1.1%)	0 (0%)	0 (0%)	0 (0%)	1 (0.6%)	1 (6.3%)	0 (0%)
Chandigarh	0 (0%)	0 (0%)	1 (1.1%)	1 (0.3%)	0 (0%)	0 (0%)	0 (0%)	0 (0%)
Dadra & Nagar Haveli and Daman & Diu	1 (0.4%)	1 (0.5%)	0 (0%)	1 (0.3%)	2 (0.5%)	0 (0%)	0 (0%)	0 (0%)
Jammu & Kashmir	6 (2.6%)	2 (1.1%)	1 (1.1%)	5 (1.6%)	13 (3.4%)	13 (8%)	0 (0%)	0 (0%)
Ladakh	2 (0.9%)	0 (0%)	0 (0%)	2 (0.6%)	0 (0%)	0 (0%)	0 (0%)	0 (0%)
Lakshadweep	0 (0%)	0 (0%)	0 (0%)	0 (0%)	1 (0.3%)	0 (0%)	0 (0%)	1 (3.1%)
NCT of Delhi	4 (1.7%)	4 (2.1%)	4 (4.3%)	3 (0.9%)	2 (0.5%)	4 (2.5%)	1 (6.3%)	0 (0%)
Puducherry	0 (0%)	2 (1.1%)	0 (0%)	1 (0.3%)	4 (1%)	0 (0%)	0 (0%)	0 (0%)
**Total**	230	190	92	318	382	162	16	32

© 2025 Jain A et al. *JAMA Network Open.*

eFigure 1. Changes in District Mean ORS Treatment and Within-District Between-Community Inequality of ORS Treatment

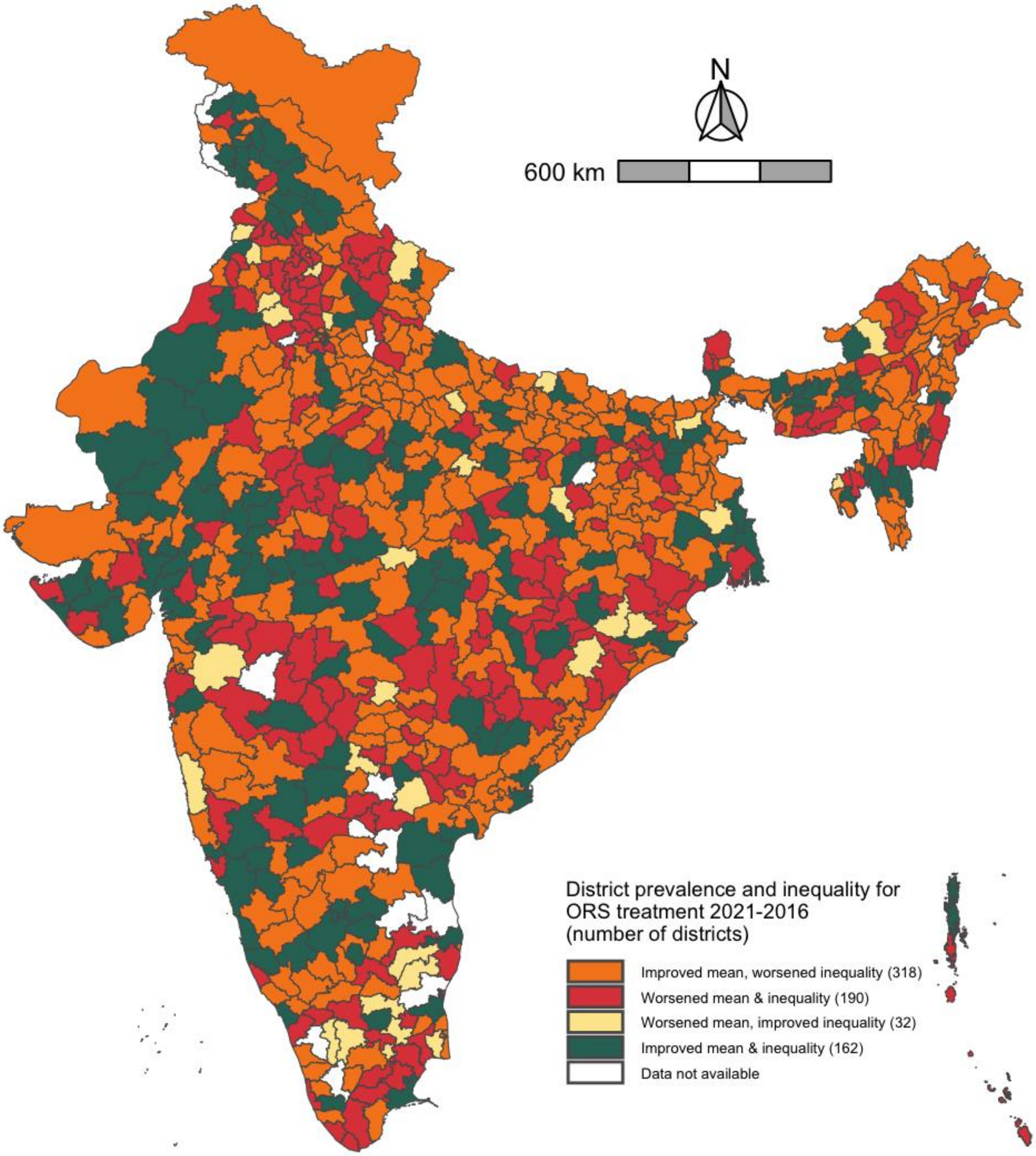

© 2025 Jain A et al. *JAMA Network Open.*

eFigure 2. Map Depicting the District-Level Correlation Between Diarrhea Prevalence and ORS Treatment in 2021.
High and low values are based on mean district values of diarrhea prevalence and ORS treatment for the survey year

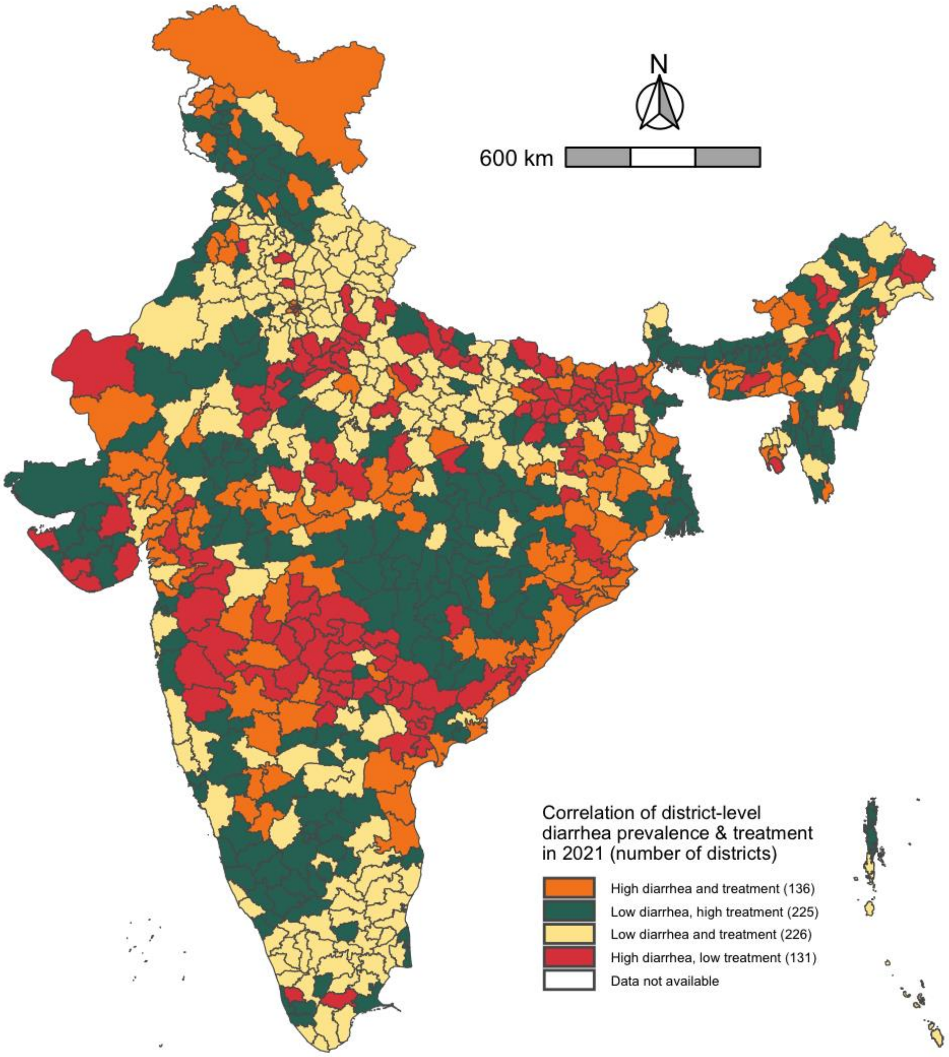

© 2025 Jain A et al. *JAMA Network Open.*

eFigure 3. Scatter Plots Describing the Correlation Between the District-Level Prevalence of Diarrhea and ORS Treatment in 2016 and 2021. The r-value is the Pearson correlation coefficient (p-value)

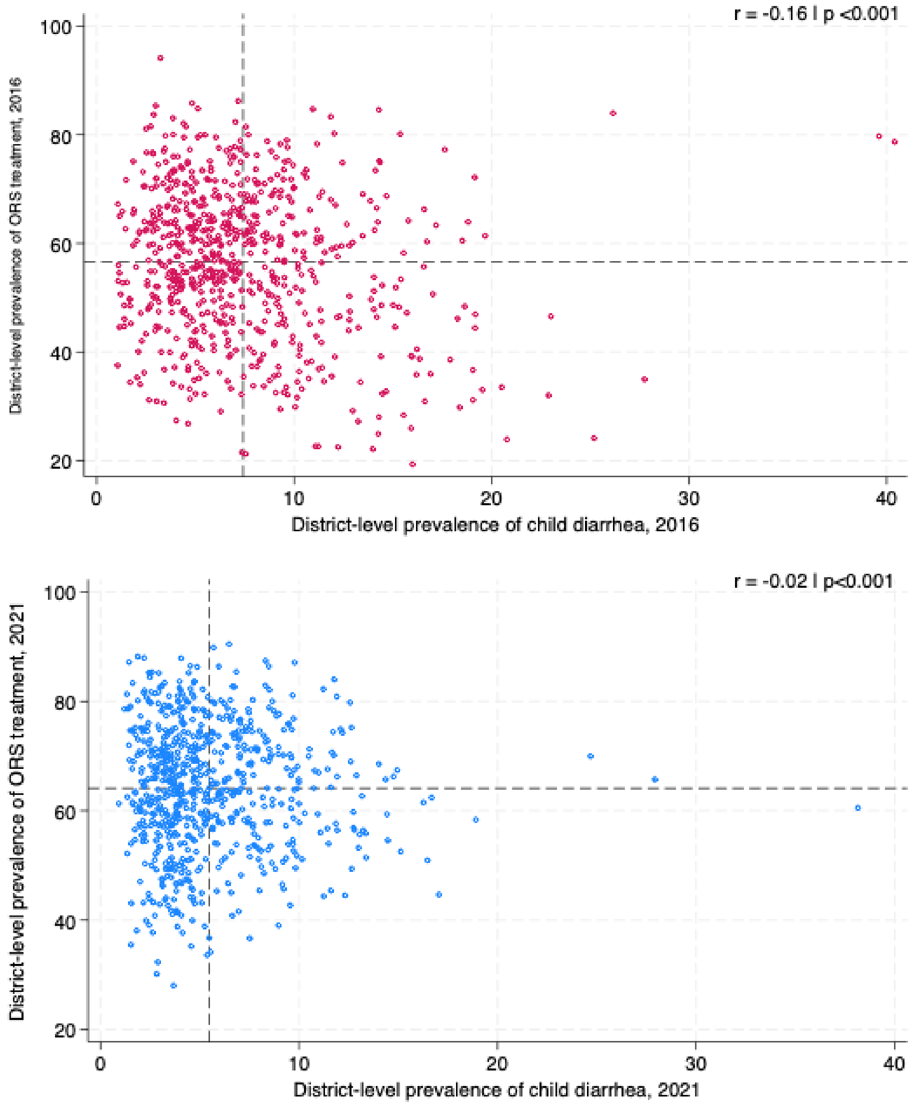

© 2025 Jain A et al. *JAMA Network Open.*

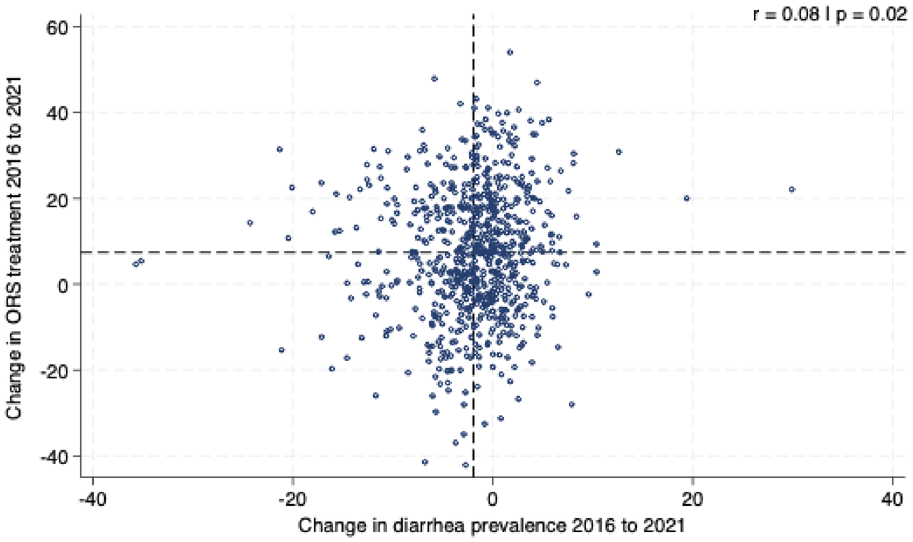

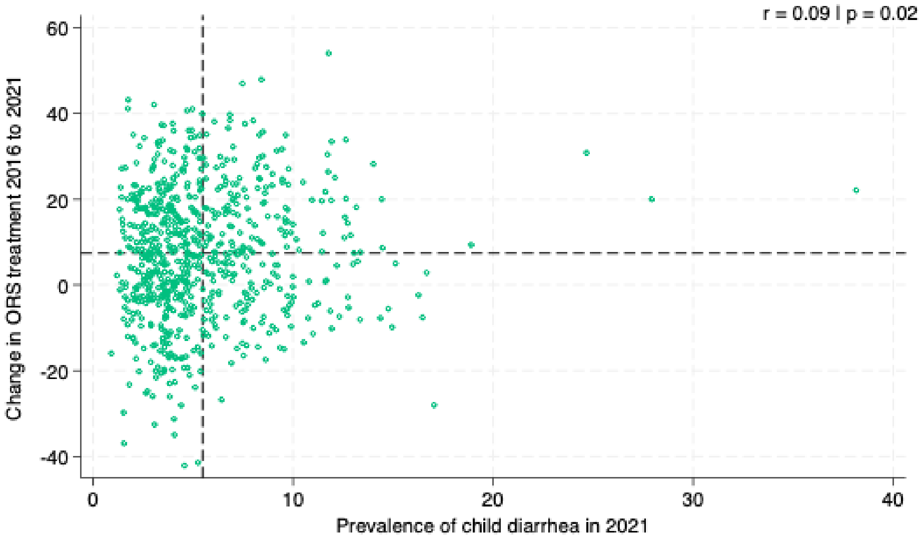

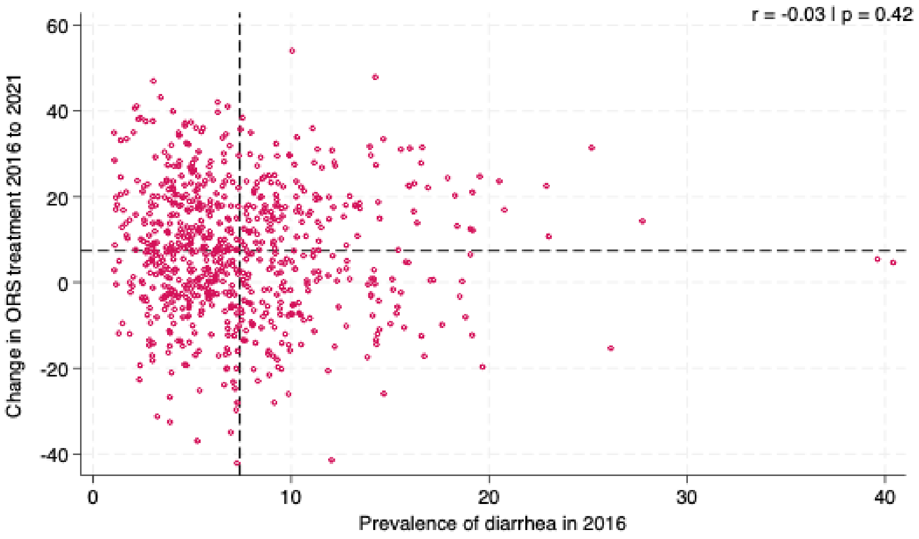

© 2025 Jain A et al. *JAMA Network Open.*
