# Supplementary material for: Prevalence and Treatment of Diarrhea Among Children in India, 2016-2021
Source: JAMA Netw Open. 2025 Aug 14;8(8):e2526979. doi: 10.1001/jamanetworkopen.2025.26979 (PMC12355290; doi:10.1001/jamanetworkopen.2025.26979)
Supplement: Supplement 2. — Data Sharing Statement [file jamanetwopen-e2526979-s002.pdf]

Data Sharing Statement

Jain. Prevalence and Treatment of Diarrhea Among Children in India, 2016-2021. *JAMA Netw Open*. Published August 14, 2025. doi:10.1001/jamanetworkopen.2025.26979

Data
Data available: No

Additional Information
Explanation for why data not available: The study is based on publicly available data and can be accessed from https://dhsprogram.com/data/available-datasets.cfm. The codes used for statistical analysis are available from the corresponding author upon reasonable request.
